# Protective Effects of Garlic and Cinnamon Oils on Hepatocellular Carcinoma in Albino Rats

**DOI:** 10.1155/2019/9895485

**Published:** 2019-10-22

**Authors:** Salah M. Aly, Hamdy A. Fetaih, Abeer A. I. Hassanin, Mosleh M. Abomughaid, Aliaa A. Ismail

**Affiliations:** ^1^Department of Pathology, Faculty of Veterinary Medicine, Suez Canal University, Ismailia, Egypt; ^2^Department of Animal Wealth Development, Faculty of Veterinary Medicine, Suez Canal University, Ismailia, Egypt; ^3^Department of Medical Laboratory Sciences, College of Applied Medical Sciences, University of Bisha, Saudi Arabia

## Abstract

Natural oils are traditional medicinal herbs, which have attracted interests for its potential anti-inflammatory and anticancer activities. The present work is aimed at evaluating the protective effect of garlic oil and cinnamon oil on diethylnitrosamine- (DENA-) and 2-acetylaminofluorene- (2-AAF-) induced p53 gene mutation and hepatocarcinogenesis in rats. Forty male albino rats were divided into 4 equal groups: control, hepatocellular carcinoma (HCC), garlic oil-HCC, and cinnamon oil-HCC. The HCC-induced group showed a significant decrease in the body mass and a significant elevation in the liver weight, alpha-fetoprotein (AFP), liver enzymes, hepatic malondialdehyde (MDA), and p53 protein expression levels as well as genetic mutations in intron 5 of p53 gene in the form of Single-Nucleotide Polymorphisms (SNPs) and insertions. In addition, the glutathione (GSH) level and superoxide dismutase (SOD) activities were increased. While HCC rats pretreated with garlic oil or cinnamon oil were significantly reversed, these destructive actions increased GSH and SOD levels. The HCC-induced group showed histopathological features of liver cancer including hypercellularity, nuclear hyperchromasia, mitotic figures, and preneoplastic foci. On the other hand, HCC rats pretreated with garlic oil or cinnamon oil revealed partial reversal of normal liver architecture. The present findings proposed that these natural oils have the ability to improve liver function, significantly reduced the liver toxicity and HCC development. However, further sophisticated studies are recommended before their use as conventional therapeutics for HCC treatment.

## 1. Introduction

Hepatocellular carcinoma (HCC) is one of the most prevalent and deadliest malignancies worldwide. It is the second leading cause of cancer-related mortalities and accounts for 70–85% of the primary malignant liver neoplasms [1]. Since a liver is mostly involved in all biochemical pathways, detoxification, and metabolic processes and it is the main site in the body that metabolizes xenobiotics, it is more prone to hepatotoxic compounds. Moreover, due to the high tolerance of the liver, HCC is hardly detected at early stage and once detected treatment has a poor prognosis [2]. Prominent risk factors associated with HCC development include cirrhosis, ingestion of aflatoxin B1-contaminated food, chronic alcohol consumption, and hepatitis B as well as C viral infections [3]. The danger of this devastating cancer is expected to increase further in coming years, due to rising incidence at alarming rates, late diagnosis, lack of definitive treatment, and poor prognosis [4]. Various genes are involved in HCC pathogenesis which can be divided into four main groups: genes regulating DNA damage response (p53), genes involved in cell cycle control (RB1, P16 INK4A, and Cyclin D), genes involved in growth inhibition and apoptosis (TGF-*β*, M6P/1GF2R, SMAD2, and SMAD4), and genes responsible for cell-cell interaction and signal transduction (APC/*β*-catenin pathway and E-cadherin) [5].

p53 gene is located on the short arm of chromosome No. 17 in human and most mammals; it encodes for a 53 kDa phosphoprotein which has the ability of binding to DNA and acting as a transcription factor. In response to chemical damage, p53 is activated to direct stress-specific transcriptional response programs such as slowing down cell division or induce programmed cell death [6, 7] which makes it a primary target for inactivation in cancer [8]. Therefore, p53 is one of the most frequently mutated genes in human cancers with the most mutations detected are Single-Nucleotide Polymorphisms (SNPs) [9]. The sites and features of DNA base changes differ among the various tumor types and depend on the carcinogen type.

A wide variety of treatment options for HCC, including chemotherapy, surgical approaches (anatomic resection and liver transplantation), ablation, transarterial chemoembolization, and systemic therapy (Sorafenib), showed limited benefits, harmful side effects, and complications [10]. Current antitumor chemotherapy still suffers from two major problems; the first is the lack of selectivity for cancer tissues, producing unwanted side effects and a broad spectrum of toxicities. The second is the multiple-drug resistance against chemotherapeutic agents [11]. The high mortality rate and associated side effects following chemotherapy and/or radiotherapy increase the demand for alternative medicine for HCC treatment. In this context, attempts have been made to find out better treatment compounds that will improve the overall prognosis of HCC and selectively target and block the tumor-specific pathways in HCC. Available plants may provide accessible and economically feasible sources of herbal HCC preventives. These natural products are believed to suppress the inflammatory process that leads to neoplastic transformation, hyperproliferation, promotion, and progression of the carcinogenic process and angiogenesis [12].

Garlic (*Allium sativum*) ranks the highest of all the herbal remedies consumed for its health benefits [13]. Garlic enhances the immune function and has antibacterial, antifungal, and antiviral activities. It is known to prevent platelet aggregation and to have hypotensive and cholesterol- and triglyceride-lowering properties. Garlic also protects against infection and inflammation and helps to lower the risk of cancer and heart disease [14]. Chemoprevention mechanisms of garlic include enhanced carcinogen detoxification, scavenging electrophiles and reactive oxygen species (ROS), increased DNA repair, decreasing inflammation, suppression of proliferation, induction of apoptosis, enhanced immunity, and inhibition of angiogenesis. Organosulfur compounds originating from garlic inhibit carcinogen activation, boost phase 2 detoxifying processes, cause cell cycle arrest mostly in the G2/M phase, and stimulate the mitochondrial apoptotic pathway [15].

Cinnamon, which belongs to the family *Lauraceae*, is widely used in traditional medicine and as a flavouring agent [16]. Cinnamon possesses significant antiallergic, antiulcerogenic, anticancer, antipyretic, antibacterial, antifungal, acaricidal, larvicidal, insulin-potentiating, antioxidant, and anti-inflammatory properties. Additionally, cinnamon is traditionally used as carminative, tonic, stomachic, and febrifuge [17].

The present study is aimed at evaluating the effect of HCC on cancer induction in albino rats through evaluation of body gain, serum biochemical analyses, tumor marker, histopathology, and immunohistochemistry as well as p53 gene mutation analyses together with investigating the possible prophylactic anticancer potential of garlic and cinnamon oils.

## 2. Material and Methods

### 2.1. Chemicals, Oils, and Reagents

Diethylnitrosamine (DENA) and 2-acetylaminofluorene (2-AAF) were purchased from Sigma-Aldrich Chemicals Co. (St. Louis, MO, USA). Garlic oil and cinnamon oil was purchased from Captain Natural Oils Company, Cairo, Egypt. Serum alpha-fetoprotein (AFP) concentrations were estimated using kits obtained from Kamiya Biomedical Company, USA. Serum biochemical parameters were performed using ready-made kits to determine alanine aminotransferase (ALT), aspartate aminotransferase (AST), alkaline phosphate (ALP), and gamma-glutamyltransferase (GGT) obtained from Randox Co., UK. Ready-made kits for reduced glutathione (GSH), superoxide dismutase (SOD), and malondialdehyde (MDA) were purchased from Biodiagnostics, Cairo, Egypt. p53 antibodies were obtained from Biotechnologies, USA.

### 2.2. Induction of Hepatocellular Carcinoma

Hepatocarcinogenesis was induced using tumor initiation and promotion model. Initiation was induced by single intraperitoneal (i.p) injection of DENA at a dose of 200 mg/kg body wt. diluted in normal saline (1 g of DENA/100 ml saline). The animals were not given any further treatment for 2 weeks as a recovery period. After this, HCC development was promoted by feeding the animals a diet containing 2-AAF which was dissolved in corn oil at a dose of 0.03% *w*/*w* of diet for 18 successive weeks [18].

### 2.3. Animals and Treatment

40 male albino rats weighing (180 ± 10 g) obtained from the Animal House of Theodor Bilharz Research Institute, Cairo, Egypt, were used for the present study. All animals were housed in standard plastic cages in the Laboratory Animal House, Faculty of Veterinary Medicine, Suez Canal University, Ismailia, Egypt. All experimental protocols were executed according to the “Guide for the Care and Use of Laboratory Animals”. After acclimating for 14 days, rats were randomly divided into four groups, each containing ten rats and treated as follows:

1. Group A (control): served as negative control rats where ten rats were orally administered 1 ml corn oil/rat for the entire period of the experiment
2. Group B (HCC-induced group): rats were treated with DENA and 2-AAF as previously explained without any other additional treatment
3. Group C (garlic oil HCC-pretreated group): rats were pretreated orally with GO (40 mg/kg BW) daily for 7 days prior to HCC induction (injection of DENA) and continue till end of the experiment [19]. Oral administration of GO continued along with concomitant administration of 2-AAF in the diet for the duration of the experiment. The body weight was measured weekly and the dosage was varied accordingly
4. Group D (cinnamon oil HCC-pretreated group): rats were pretreated orally with cinnamon oil (100 mg/kg BW) daily for 7 days before HCC induction (injection of DENA) and continue till end of the experiment [20]. Oral administration of cinnamon oil continued along with concomitant administration of 2-AAF in the diet for the duration of the experiment. The body weight was measured weekly and the dosage was varied accordingly

### 2.4. Sampling

At the end of the study (at 21^th^ week), serum samples and tissue specimens were collected from all groups. Animals were subjected to mild ether anesthesia, and blood samples were collected from retroorbital venous plexus in plain centrifuge tubes by a clean sterile capillary tube that was inserted at the inner canthus of the eye. The blood was left for 20 minutes to coagulate then centrifuged at 3000 rpm for 15 minutes to separate the serum; then, serum was stored at -20°C to be used for AFP level detection and biochemical analysis. After blood collection, rats were dissected and the postmortem findings were recorded; then, samples from the internal organs (liver, lung, intestine, and kidney) were taken. The livers were excised, washed with ice-cold phosphate buffer saline (PBS) to remove any extraneous matter and to observe tumor morphology, blotted to dry, and weighed, and small sections were cut. The first liver section was used for the preparation of the liver tissue homogenate to be used for the antioxidant state analysis. The second liver section was fixed in 10% neutral buffered formalin for the histopathological examination and p53 immunohistochemistry. The last section was immediately stored at -20°C for DNA extraction.

### 2.5. Parameters Measured

The body weight, absolute liver weight, and gross findings were evaluated in all groups. The AFP level in serum of rat blood was determined at 450 nm by using the enzyme-linked immunosorbent assay (ELISA) test kit. Serum parameters ALT, AST, ALP, and GGT were measured using respective kits according to the manufacturer's instructions. The hepatic GSH content and SOD activity were estimated, and lipid peroxidation was measured in the liver tissue as the MDA level according to the method of Elguindy et al. [21].

### 2.6. Histopathological Examination

Tissue specimens from the liver, lung, intestine, and kidney were freshly taken and fixed in 10% neutral buffered formalin, washed in running tap water, dehydrated in ascending concentration gradients of ethyl alcohol, cleared in two changes of xylene, then embedded in paraffin wax, and afterwards sliced into thin sections of 5 *μ*m thickness using a rotatory microtome. Sections were stained with H&E stain according to the standard method [22] then examined microscopically.

### 2.7. Histopathological Lesion Scoring

The bile duct hyperplasia and other degenerative changes were graded, according to Patial et al. [22], with a score as follows: Score 1: occasional/in few places (mild), Score 2: fairly common/multifocal areas (moderate), and Score 3: diffuse (severe). A mitotic index was also calculated in the liver by counting the mitotic bodies per ten microscopic fields per animal according to Patial et al. [22].

### 2.8. Immunohistochemistry of p53

The paraffin-embedded livers were sectioned into 5 *μ*m thickness and mounted on positively charged slides for p53 immunohistochemistry according to the manufacturer's instructions. Sections were deparaffinized in xylene and rehydrated with a descending series of alcohol solution, then washed with water. The sections were incubated with the primary antibody for p53 (Cat. No. 10-0001; Genemed Biotechnologies, USA) at a dilution of 1 : 500, for 30-60 minutes at room temperature. The slides were rinsed three times with Wash Buffer for 2 minutes each. Poly-HRP (horseradish peroxidase) reagent A was applied to tissue sections and coincubated for 15 ± 1 min. Then, the slides were washed three times with Wash Buffer (PBS) for 2 minutes each. The reaction was visualized/observed through the use of DAB substrate chromogen buffer solution to the tissues and incubated 5-10 minutes in room temperature followed by washing with tap water to remove excess substrate solution. For quantitative analysis, the intensity of immunoreactive parts was used as a criterion of cellular activity after subtracting background noise. Measurement was done using an image analyzer (ImageJ program). From each slide of all experimental groups, 9 fields were randomly selected. The total field and immunohistochemial (IHC) stained areas (reactions) were calculated, and the percentage of the IHC stained area was calculated as follows: IHC stained area% = IHC stained area/total area × 100.

### 2.9. PCR Amplification of Partial p53 Gene Sequence Representing Exons 5-7

#### 2.9.1. DNA Extraction

Total genomic DNA of liver samples was extracted using the conventional phenol/chloroform method (Sambrook and Russell, 2001) as follows: 200 *μ*l of homogenized tissue suspension was mixed with 700 *μ*l of lysis buffer (50 mM Tris-HCl pH 8.0, 200 mM NaCl, 20 mM EDTA pH 8.0, and 1% SDS) and 20 *μ*l of proteinase K was added. The mixture was then incubated at 56°C for an hour during which the tube was inverted 3-5 times every 20 minutes. 600 *μ*l of phenol/chloroform/isoamyl alcohol (25 : 24 : 1) solution was added followed by centrifugation for 10 minutes at 12.000 rpm. The aqueous phase was transferred to a new microcentrifuge tube, and DNA was precipitated by adding 600 *μ*l of absolute ethanol in the presence of a high concentration of salts (30 *μ*l of 3 M sodium acetate (pH 5.2). The precipitated DNA was washed in 500 *μ*l of 70% ethanol, air dried, and subsequently dissolved in 100 *μ*l of TE buffer pH 7.6 (10 mM Tris and 1 mM EDTA).

#### 2.9.2. Oligonucleotide Primers, PCR, and Agarose Gel Electrophoresis

Six primers, one sense (F), and one antisense (R) primer for each of the three exons (exon 5, exon 6, and exon 7) of rat p53 gene were used (Table 1). PCR reactions were done in a total reaction volume of 50 *μ*l each, containing 5 *μ*l of DNA template, 1 *μ*l forward primer, 1 *μ*l reverse primer, 25 *μ*l PCR master mix, and 18 *μ*l ddH_2_O. Amplification was done using a Little Genius BIOER thermal cycler under the following conditions: predenaturation step at 94°C for 3 minutes, followed by 30 cycles of denaturation for 30 seconds at 94°C, annealing for 30 seconds at 60°C, and extension for 30 seconds at 72°C, with a final extension step at 72°C for 3 minutes. Amplification was verified by electrophoresis on 1% (*w*/*v*) agarose gel in 1x TAE (Tris acetic acid EDTA) buffer, using a 100 bp DNA ladder as a molecular weight marker for confirmation of the PCR product length. Agarose gel was stained with ethidium bromide (1 *μ*g/ml), visualized under a UV Transilluminator, and photographed.

### 2.10. DNA Sequencing and Single-Nucleotide Polymorphism (SNP) Analysis

PCR products from both control and HCC-induced groups were sequenced. All loci were directly sequenced in both forward and reverse directions using the same primers used for PCR amplification by dye terminator cycle sequencing technique. Sequence alignment of the resulted sequences with the corresponding functional p53 gene sequence available in GenBank to locate sequence variants in DNA and protein sequences were done by the CLUSTAL W method using the Lasergene MegAlign program (version 15.1, DNASTAR Inc.).

### 2.11. Statistical Analysis

Statistical analyses were performed using software SPSS version 20.0. One-way analysis of variance (ANOVA) was used to detect the significant changes between the groups followed by Duncan's multiple comparison test to find if there was any significant difference between groups. Statistical significance was considered when *P* ≤ 0.05. All the values were expressed as mean ± SE (standard error of the mean).

## 3. Results

**Figure 1:**
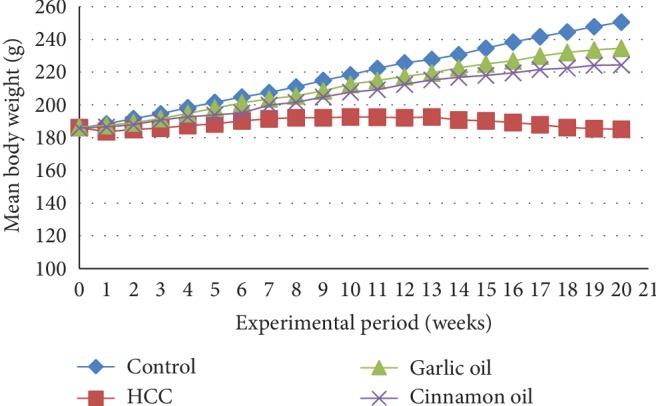
Body weight of rats in different experimental groups throughout the weeks of experiment.

On the first 2 weeks, the HCC-induced group showed a slight decrease in the body weight then began to show a very slow increase in the growth rate from 3^rd^ to 14^th^ weeks. Afterwards, it began to decrease gradually, in a time-dependent manner, from 14^th^ week till the end of the experiment. At the end of 21 weeks, the body weight of rats in the control group was increased to about 1.35-fold of the initial value, while that of rats in the HCC-induced group was decreased by 0.5%. In contrast, the body weight of HCC rats pretreated with garlic oil or cinnamon oil was significantly recovered where the body weight gain was increased to 1.26-fold and 1.21-fold, respectively. The absolute liver weight in the HCC-induced group was increased compared with that in the control group. Administration of garlic oil or cinnamon oil significantly reduced the liver weight as compared with the HCC-induced group (Figure 1 and Table 2).

**Figure 2:**
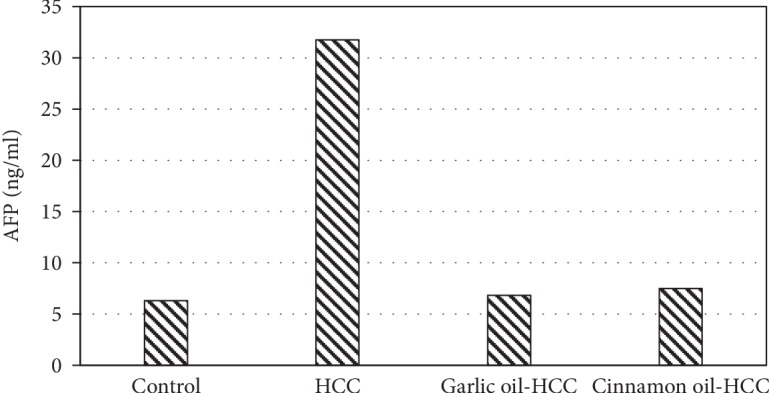
Tumor marker alpha fetoprotein (AFP) levels in serum of experimented rats.

The serum AFP level is the most common predictor for HCC. The results showed that garlic oil or cinnamon oil could decrease the serum AFP level, which was significantly increased in the HCC-induced group (Figure 2). The activities of serum ALT, AST, ALP, and GGT were markedly increased in the HCC-induced group compared with the control group but were significantly decreased by garlic oil or cinnamon oil (Table 3). DENA/2-AAF significantly increased the MDA level, which was markedly suppressed by garlic oil or cinnamon oil treatment. Furthermore, DENA/2-AAF caused significant decreases in the level of GSH and impaired the activities of SOD. Compared with the HCC-induced group, garlic oil or cinnamon oil significantly increased the hepatic level of GSH and the activities of SOD (Table 4).

### 3.1. Gross Finding of the Liver

**Figure 3:**
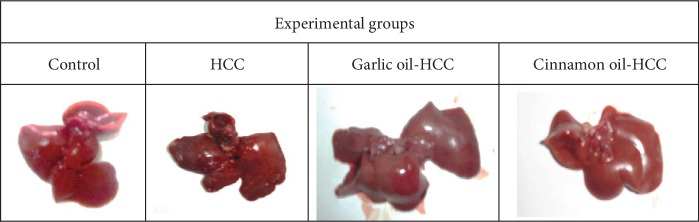
A gross liver of experimental groups, control rats showing normal liver, HCC-induced rats showing that grayish-white pin point foci with variable sizes gave the external surface of the liver a rough, nodular, and irregular appearance, garlic oil-HCC rats showing nearly normal liver size and morphology, and cinnamon oil-HCC rats showing nearly normal liver size and morphology.

HCC-induced rats revealed severe enlargement and swelling of the liver with rough and rounded edges, increased friability of the parenchyma, dark red color, and developed dysplastic nodules which appeared as grayish-white visible pin point foci with variable sizes measuring from 1 mm to 5 mm in diameter that gave the external surface of the liver a rough, nodular, and irregular appearance. The number of nodules was analyzed: compared with the HCC-induced group; garlic oil or cinnamon oil significantly reduced the number of nodules (Figure 3). A significant reduction in liver enlargement was observed in HCC rats pretreated with garlic oil or cinnamon oil compared with the HCC-induced group.

### 3.2. Histopathology

**Figure 4:**
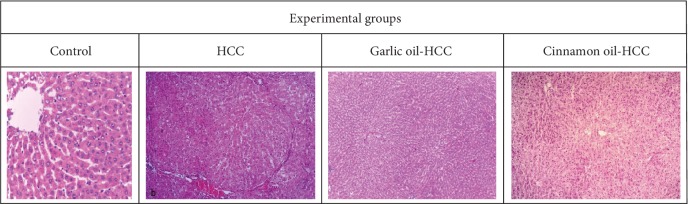
Liver tissue in each group of rats (H&E staining, 200), control rats showing a normal hexagonal lobular pattern of hepatic tissue, HCC-induced rats showing that delicate fibrous connective tissue bands cause distortion of the normal architectural pattern of the hepatic parenchyma, garlic oil-HCC rats showing nearly preserved architecture, and cinnamon oil-HCC rats showing partial reversal of normal liver architecture.

Liver sections from control rats revealed a normal hexagonal lobular pattern of the hepatic tissue with normal size and shape of hepatocytes. However, liver tissues from the HCC-induced group exhibited disordered architecture with large number of pseudolobules (dysplastic nodules/early neoplastic nodules) which exert compression and pressure atrophy on the adjacent normal parenchyma, infiltration of inflammatory cells, and pleomorphic abnormal cells with the irregular-shaped cytoplasm and enlarged hyperchromatic nuclei. Varying degrees of nuclear atypia were detected with increased number of figures (binucleated cells). Portal areas are enlarged with bile duct proliferation and massive mononuclear inflammatory cell infiltration. The liver of garlic oil-HCC-pretreated rats showed a noticeable improvement where the architecture was nearly preserved with minimal mononuclear inflammatory infiltration. The histopathological examination of the liver of the cinnamon oil-HCC-pretreated group revealed marked recovery of hepatic architecture with mild hepatic degeneration and no nodules detected (Figure 4).

The internal organs (intestine, kidneys, and lungs) of experimented rats did not show any histopathological alterations in the control group. HCC-induced rats showed marked degeneration, focal necrosis, congestion, and moderate to massive mononuclear cell infiltrations. HCC-induced rats pretreated with garlic oil or cinnamon oil showed mild degeneration and focal mononuclear cell infiltration.

### 3.3. Histopathological Lesion Scoring

The intensity of hepatic degenerative changes, bile duct hyperplasia, and the mitotic index were significantly increased in the HCC-induced group. The garlic or cinnamon oil-HCC-pretreated groups demonstrated a significant decrease in these parameters compared with the HCC-induced group (Table 5).

### 3.4. Immunohistochemistry (IHC) of p53

**Figure 5:**
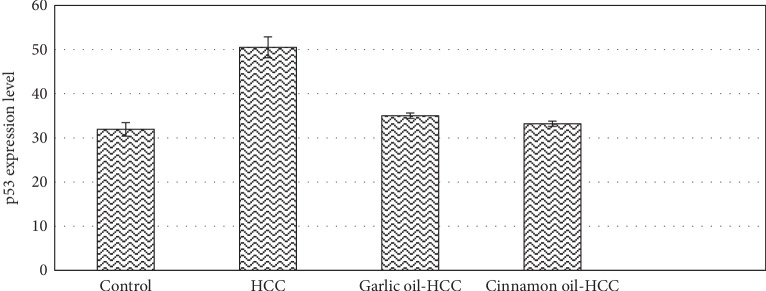
p53 expression levels (IHC) of different studied experimental groups at 3 and 5 months of the experiment.

Rats of the HCC-induced group revealed a significant increase in p53 expression levels in liver tissues compared to the control one. p53 expression levels in the liver tissues were significantly decreased in both HCC-pretreated groups when compared with the HCC-induced group (Figure 5).

### 3.5. PCR Amplification Results of Rat p53 Gene Exons 5, 6, and 7

**Figure 6:**
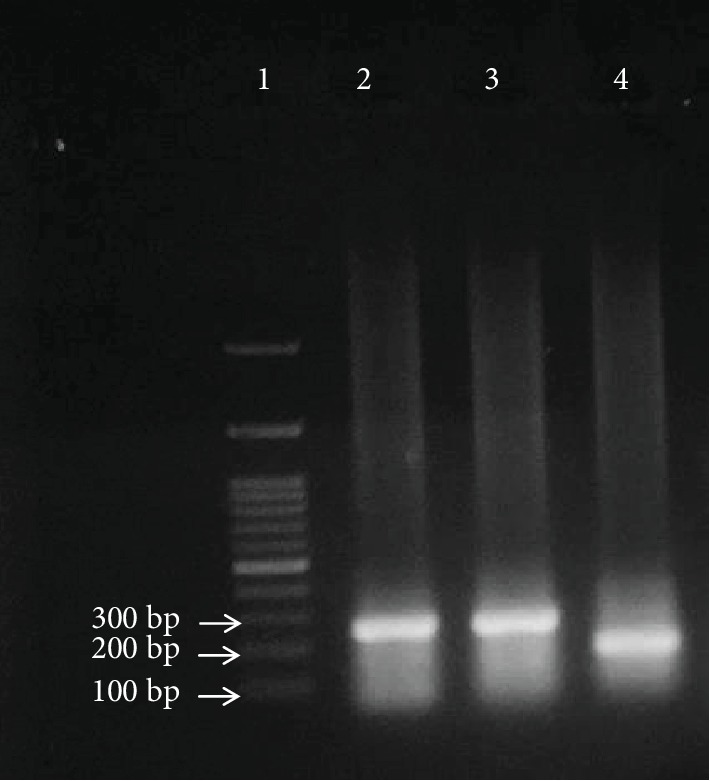
PCR amplification results of p53 gene exons 5, 6, and 7 in the control group. Lane 1: 100 bp DNA ladder. Lane 2: 265 bp PCR amplicon representing a part of intron 4, exon 5, and part of intron 5 of rat p53 gene. Lane 3: 273 bp PCR amplicon representing a part of intron 5, exon 6, and part of intron 6 of rat p53 gene. Lane 4: 188 bp PCR amplicon representing a part of intron 6, exon 7, and part of intron 7 of rat p53 gene.

**Figure 7:**
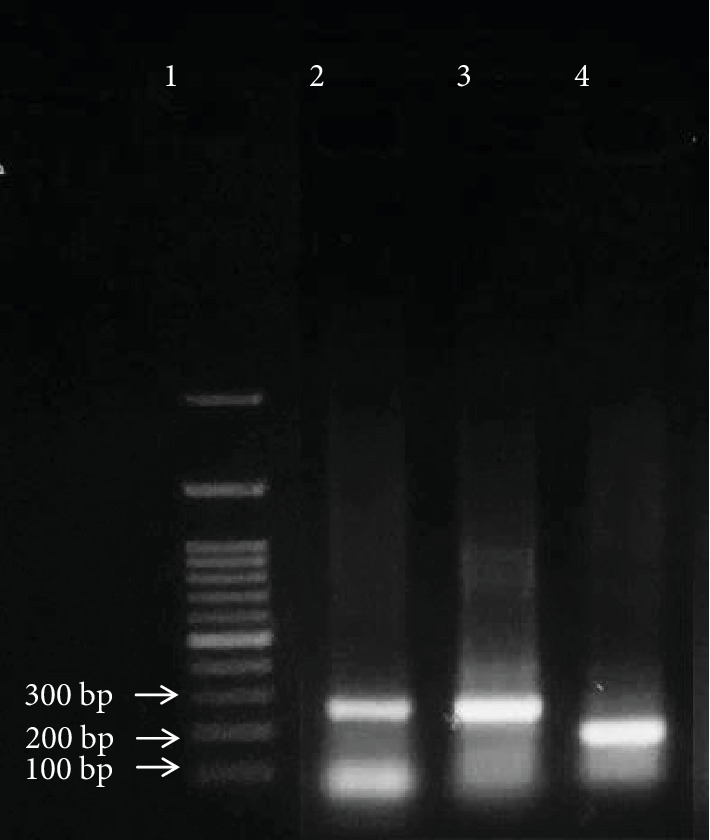
PCR amplification results of p53 gene exons 5, 6, and 7 in the HCC-induced group. Lane 1: 100 bp DNA ladder. Lane 2: 265 bp PCR amplicon representing a part of intron 4, exon 5, and part of intron 5 of rat p53 gene. Lane 3: 275 bp PCR amplicon representing a part of intron 5, exon 6, and part of intron 6 of rat p53 gene. Lane 4: 188 bp PCR amplicon representing a part of intron 6, exon 7, and part of intron 7 of rat p53 gene.

The amplified PCR products of rat p53 gene exons 5, 6, and 7 in both control and HCC-induced groups under the study are shown in Figures 6 and 7, respectively.

### 3.6. Sequence Analysis of the PCR Amplicons Representing Exons 5, 6, and 7 of Rat p53 Gene in Both Control and HCC-Induced Groups

#### 3.6.1. Sequence Analysis of the PCR Amplicon Representing Exon 5 of Rat p53 gene in Both Control and HCC-Induced Groups

**Figure 8:**
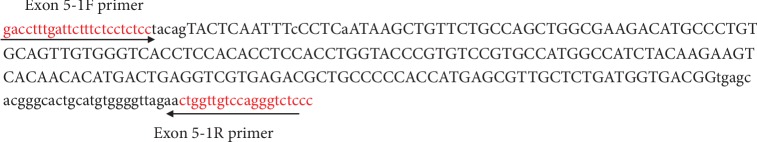
Nucleotide sequence of the amplified PCR product (265 bp) representing exon 5 of rat p53 gene in the control group.

**Figure 9:**
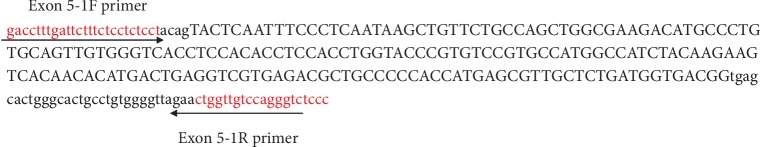
Nucleotide sequence of the amplified PCR product (265 bp) representing exon 5 of rat p53 gene in the HCC-induced group.

In both control and HCC-induced rat groups, Exon 5-1F and Exon 5-1R primers were annealed successfully at 1058 bp-1081 bp location at intron four and 1302 bp-1321 bp location at intron five of rat p53 gene, respectively. These two primers resulted in the amplification of approximately 265 bp PCR product at nucleotide position 1058-1321 bp of p53 gene. Uppercase is the target fragment in the amplification process (exon five; 185 bp); lowercase before exon five nucleotide sequence represents the part of intron four (29 bp), while the lowercase after exon five nucleotide sequence represents the part of intron five (51 bp) amplified using Exon 5-1F and Exon 5-1R primer pair. Positions of both primer pair are underlined (Figures 8 and 9).

#### 3.6.2. Sequence Analysis of PCR Amplicon Representing Exon 6 of Rat p53 Gene in Both Control and HCC-Induced Groups

**Figure 10:**
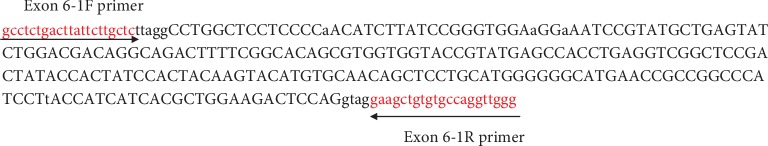
Nucleotide sequence of the amplified PCR product (273 bp) representing exon 6 of rat p53 gene in the control group.

**Figure 11:**
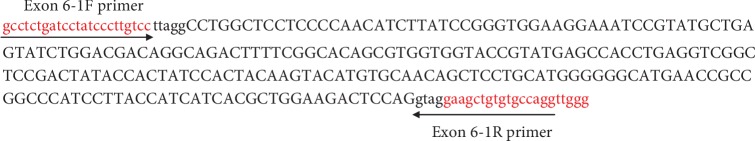
Nucleotide sequence of the amplified PCR product (275 bp) representing exon 6 of rat p53 gene in the HCC-induced group.

In both control and HCC-induced rat groups, Exon 6-1F and Exon 6-1R primers were annealed successfully at 1323 bp-1343 bp location at intron five and 1575 bp-1595 bp location at intron six of rat p53 gene, respectively. These two primers resulted in amplification of approximately 273 bp and 275 bp PCR products in both control and cancer-induced groups, respectively, at nucleotide position 1323-1343 bp of p53 gene. Uppercase is the target fragment in the amplification process (exon six; 222 bp). Lowercase before exon six nucleotide sequence represents the part of intron five (26 bp in the control group and 28 bp in the cancer-induced group), while the lowercase after exon six nucleotide sequence represents the part of intron six (25 bp) amplified using Exon 6-1F and Exon 6-1R primer pair. Positions of both primer pair are underlined (Figures 10 and 11).

#### 3.6.3. Sequence Analysis of PCR Amplicon Representing Exon 7 of Rat p53 Gene in Both Control and HCC-Induced Groups

**Figure 12:**
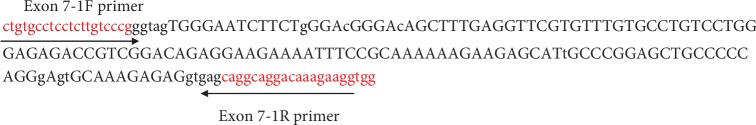
Nucleotide sequence of the amplified PCR product (188 bp) representing exon 7 of rat p53 gene in the control group.

**Figure 13:**
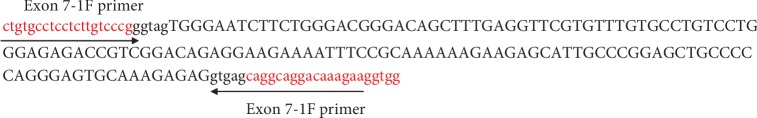
Nucleotide sequence of the amplified PCR product (188 bp) representing exon 7 of rat p53 gene in the HCC-induced group.

In both control and HCC-induced rat groups, Exon 7-1F and Exon 7-1R primers were annealed successfully at 1605 bp-1624 bp location at intron six and 1772 bp-1792 bp location at intron seven of rat p53 gene, respectively. These two primers resulted in amplification of approximately 188 bp PCR product at nucleotide position 1605-1792 bp of p53 gene. Uppercase is the target fragment in the amplification process (exon seven; 137 bp); lowercase before exon seven nucleotide sequence represents the part of intron six (25 bp) while the lowercase after exon seven nucleotide sequence represents the part of intron seven (26 bp) amplified using Exon 7-1F and Exon 7-1R primer pair. Positions of both primer pair are underlined (Figures 12 and 13).

### 3.7. Sequence Alignment Results of PCR Amplicons Representing Exons 5, 6, and 7 of Rat p53 Gene in Both Control and HCC-Induced Groups

Sequence alignment of PCR amplicons representing exons 5, 6, and 7 of rat p53 gene in both control and HCC-induced groups under the study was done by the CLUSTAL W method using the Lasergene MegAlign program (Ver 15.1, DNASTAR Inc.).

#### 3.7.1. Sequence Alignment Results of PCR Amplicon Representing Exon 5 of Rat p53 Gene in Both Control and HCC-Induced Groups

**Figure 14:**
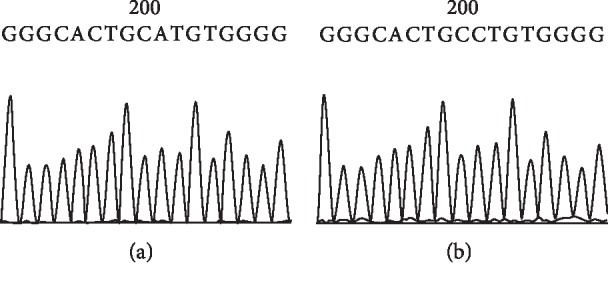
Direct sequencing of the amplified PCR product (265 bp) representing exon 5 of rat p53 gene in both control (a) and HCC-induced (b) groups showing an A to C transversion.

**Figure 15:**
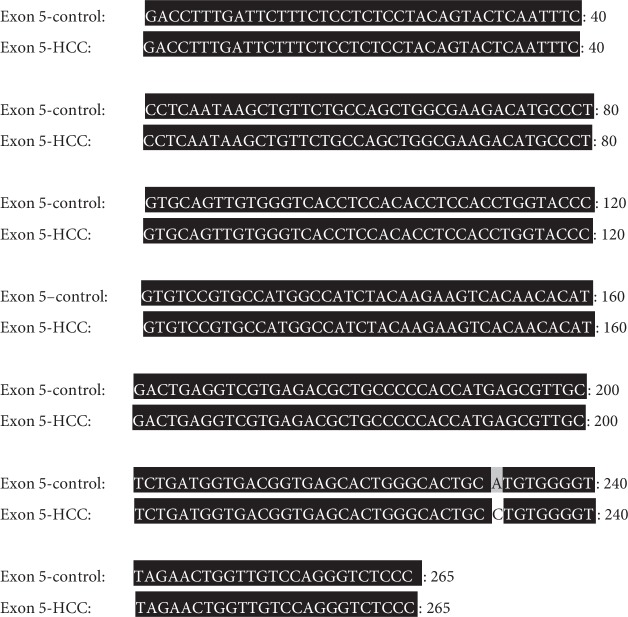
Nucleotide sequence alignment of the amplified PCR product (265 bp) representing exon 5 of rat p53 gene in both control and HCC-induced groups showing an A to C transversion at position 1289 of intron 5.

**Figure 16:**
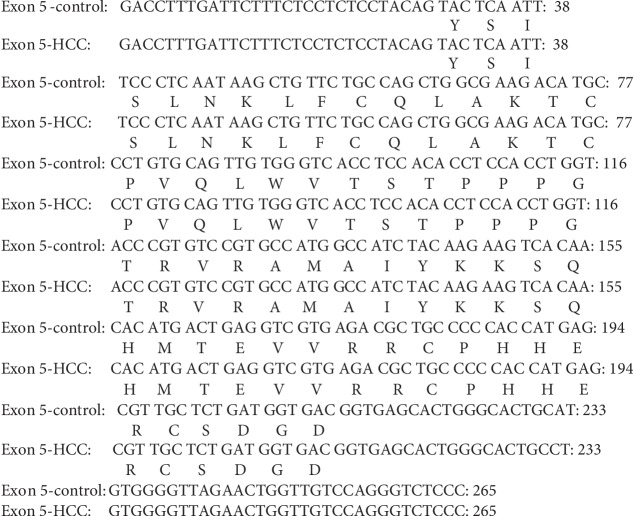
Amino acid sequence alignment of rat p53 gene, exon 5 in both control and HCC-induced groups showing complete homogeneity.

Sequence alignment of PCR amplicon representing exon 5 of p53 gene showed an A to C transversion at position 1289 of intron 5 (A 1289 C) (Figures 14, 15, and 16).

#### 3.7.2. Sequence Alignment Results of PCR Amplicon Representing Exon 6 of Rat p53 Gene in Both Control and Cancer-Induced Groups

**Figure 17:**
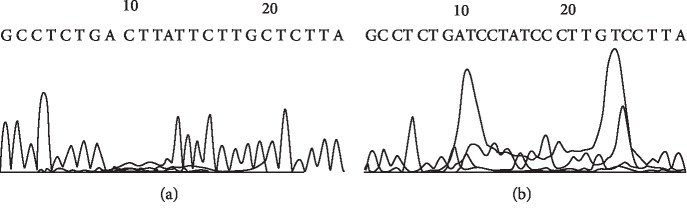
Direct sequencing of the amplified PCR product representing exon 6 of rat p53 gene in both control (a) and HCC-induced (b) groups showing two insertion mutations of the nucleotides T and C, three T to C transitions, and one C to T transition.

**Figure 18:**
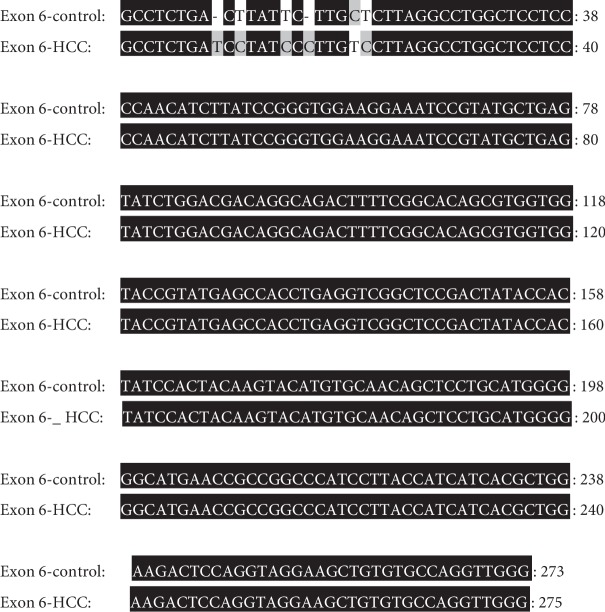
Nucleotide sequence alignment of the amplified PCR product representing exon 6 of rat p53 gene in both control and cancer-induced groups showing two insertion mutations of the nucleotides T and C at position numbers 1331 and 1338 of intron 5, respectively, three T to C transitions at positions 1332 (T 1332 C), 1336 (T 1336 C), and 1342 (T 1342 C), respectively, of intron 5, and one C to T transition at position 1341 (C 1341 T) of intron 5.

**Figure 19:**
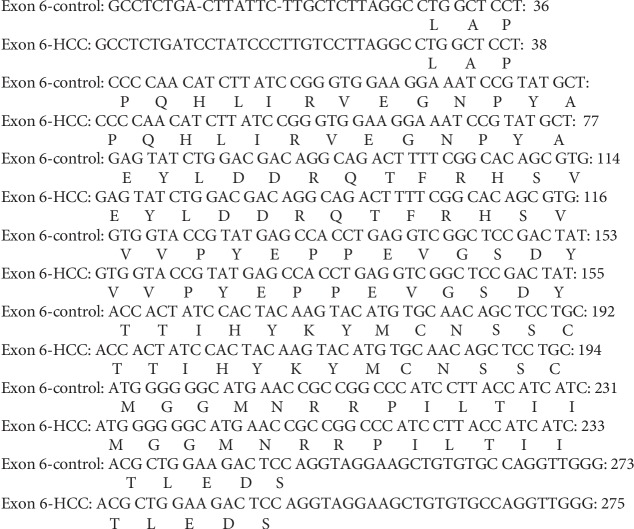
Amino acid sequence alignment of rat p53 gene, exon 6 in both control and cancer-induced groups showing complete homogeneity.

Sequence alignment of PCR amplicon representing exon 6 of p53 gene showed two insertion mutations of the nucleotides T and C at positions number 1331 and 1338 of intron 5, respectively, three T to C transitions at positions 1332 (T 1332 C), 1336 (T 1336 C), and 1342 (T 1342 C), respectively, of intron 5, and one C to T transition at position 1341 (C 1341 T) of intron 5 (Figures 17, 18, and 19).

#### 3.7.3. Sequence Alignment Results of PCR Amplicon Representing Exon 7 of Rat p53 Gene in Both Control and Cancer-Induced Groups

**Figure 20:**
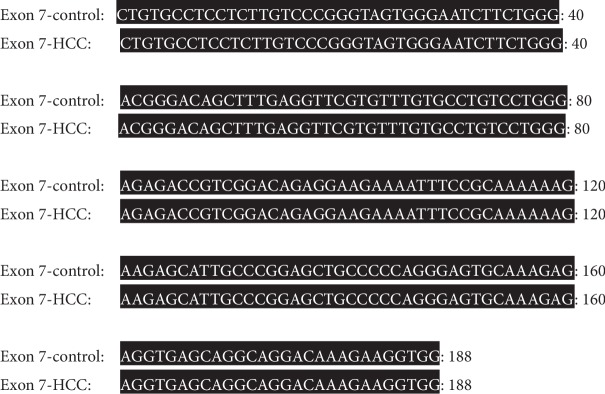
Nucleotide sequence alignment of the amplified PCR product (188 bp) representing exon 7 of rat p53 gene in both control and cancer-induced groups showing complete homogeneity.

**Figure 21:**
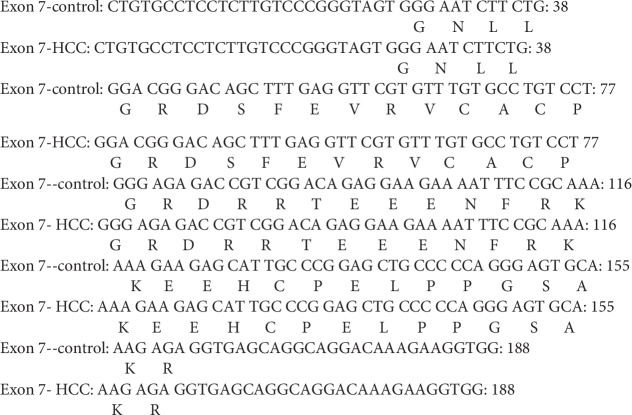
Amino acid sequence alignment of rat p53 gene, exon 7 in both control and cancer-induced groups showing complete homogeneity.

Sequence alignment of PCR amplicon representing exon 7 of p53 gene showed complete homogeneity between the two groups under the study. No SNPs were located (Figures 20 and 21).

## 4. Discussion

In the present study, the chemoprotective effects of garlic oil (40 mg/kg) and cinnamon oil (100 mg/kg) on liver tumorigenesis in male albino rats were evaluated. In this study, there was a progressive loss in body weight of HCC-induced rats throughout the experiment duration and this reduction in body weight correlated well with decreased food intake. Anorexia, oligophagia, impaired digestion and absorption, abnormal hepatic function upon exposure to hepatocarcinogens, tumor cell competition for nutrients, and increased energy expenditure may contribute to this weight loss and eventually to the development of cachexia which is the hallmark of cancer (Schneider et al., 2017). The steadily increase in the body weight of garlic oil and cinnamon oil HCC-pretreated groups may result from increased appetite and food consumption, improved intestinal villi health, and increased digestion and absorption of nutrients. This increase in the body weight proves that garlic and cinnamon oils able to stop the withering effects on HCC. Concerning the increase in the absolute liver weight that observed in rats with HCC can be potentially attributed to the formation of neoplastic/dysplastic nodules and/or hepatocyte hyperplasia and hypertrophy. However, garlic and cinnamon oils were found to suppress the neoplastic nodule formation effectively and reduce the absolute liver weight, showing their anticancer effectiveness. Taha et al. [23] and Seydi et al. [24] proved that DENA and 2-AAF triggered an appreciable reduction in the body weight and increase in the liver weight during HCC induction in experimental rats. The results of increased body weight gain in garlic oil HCC-pretreated animals compared to the HCC group came in agreement with Zhang et al. [19] and Eidi et al. [25]. Our results showed a significant rise in the AFP levels in HCC-induced rats which may have resulted from DENA/2-AAF hepatic intoxication that caused alterations in the hepatocytes and increased transcription of AFP gene or posttranslational modification affecting AFP production. In the present investigation, the AFP levels were downregulated or normalized in HCC-induced rats in response to garlic oil and cinnamon oil pretreatment possibly due to the inhibition of transcription of AFP gene by these natural agents and their hepatotoxic preventing effect. These detoxifying effects of garlic and cinnamon oils are related to their ability to bind to exogenous toxins and the induction of phase II antioxidant enzymes. Regarding the serum biochemical analyses, we observed an elevation in serum ALT, AST, ALP, and GGT in DENA/2-AAF-treated rats. This augmentation may be due to hepatic necrosis and subsequent leakage of these enzymes from neoplastic cells or irreversibly damaged hepatic cells into circulation or may be due to possible effect of tumor on remote tissues leading to loss of its enzyme content and their released into the blood [26]. These findings go in agreement with the results of the current histopathological study which revealed multiple-variable-sized vacuoles in the hepatocytes, suggesting different stages of steatosis, multifocal areas of necrosis of HCC-bearing rats. On the other hand, we noticed that cinnamon oil and garlic oil significantly recouped the enhanced levels of these enzymes back to normal. These observations might be due to the presence of hepatoprotective natural bioactive constituents in the oily extract of garlic and cinnamon which have the ability to reduce free radical-induced hepatic damage. This indicates that these compounds helped in hepatocyte regeneration, improved the hepatic structure and function, prevent liver damage, halted further liver parenchymal damage, and maintained cell membrane stabilization/integrity, therefore suppressed the enzyme release. These results are in support with the previous studies of Medhat et al. [27], Sawada et al. [28], and Shaarawy et al. [29]. In the present investigation, GSH and SOD levels were decreased in HCC-induced animals. This depletion may be caused by the increase in free radical generation from metabolism of carcinogenic chemicals used and increased lipid peroxidation as well as decreased expression of these antioxidant enzymes during hepatocellular damage [30]. In garlic and cinnamon oils, HCC-pretreated rats' higher levels of hepatic GSH and SOD noticed may be due to suppression of lipid peroxidation and protein oxidation, inhibition of ROS production consistent with radical scavenging activity, and reduced DNA carcinogen interaction indicating the restoration of the antioxidant system of the liver [31]. The level of MDA increased in HCC-induced rats indicating for a high level of lipid peroxidation which is in turn a marker of cell membrane damage, alterations in structure and function of cellular membranes, and failure of antioxidant defence mechanisms to prevent the formation of excessive free radical as previously reported by Taha et al. [23], Elguindy et al. [21], and Kadasa et al. [32]. However, MDA levels were restored back to normal in the garlic oil-HCC-pretreated group indicating that the effect of garlic oil was greater than cinnamon oil in decreasing LPO. This could be presumably related to their ability to scavenge reactive oxygen species especially the most active hydroxyl and peroxyl radicals that initiated lipid peroxidation and counteract the oxidative step which is decisive in avoiding the development of HCC. Thus, the present investigation suggested that garlic and cinnamon oils would exert a chemoprotective effect by reversing the oxidant-antioxidant imbalance during hepatocarcinogenesis induced by DENA and 2-AAF. To verify the anticancer activity of garlic and cinnamon oils, histopathological studies were carried out. Marked changes were observed in the architecture of the liver of HCC-bearing animals where the characteristic histological features of the normal liver tissue were disorganized or lost. This could be attributed to the chain of events starting with oxidative stress leading to membrane damage, inflammatory cell infiltration, cellular injury, cellular and nuclear atypia, and other morphological features during transformation into HCC by DENA and 2-AAF. All these changes may have contributed to the malignant hepatocyte growth and progression assuring the induction of HCC. DENA is known to cause enlargement of the nucleus of hepatocytes, decreased glycogen content, centrilobular necrosis, and macrophage and neutrophil infiltrations in the liver tissue. The promotion of liver tumors by 2-AAF is accompanied by cirrhosis, and malignant cells in the liver tissue are best described as hyperplastic nodules. 2-AAF gives an advantage for proliferation to the initiated cells. Many of these nodules give rise to distinct HCC in clinical follow-up studies and are therefore considered precancerous lesions. Nuclear changes (mitoses) explain the presence of different cell populations and genetic anomalies, which occur during DNA replication and cell division phases. Nevertheless, pretreatment with garlic and cinnamon oils to HCC rats not only reduced the adverse effects of DENA/2-AAF but also helped in partial to complete restoration of histological features of the liver, alleviated the percentage of nodule incidence, and recovered the hepatic damage. An elevated mitotic index was observed in the HCC-induced group revealing increased cell proliferation, whereas in both HCC-pretreated rats, mitotic arrest showed the ability of garlic and cinnamon oils to modulate DNA damage.

Further, to substantiate the proapoptotic action of garlic and cinnamon oils at a cellular level, we performed IHC for p53 on liver tissue sections. The IHC analysis revealed that p53 protein expression in the liver tissues was the highest in the HCC-induced group. Overexpression of proapoptotic protein p53 in the HCC-induced group could be produced in response to hepatocyte inflammation and liver steatosis. The tumor suppressor gene p53 is expressed in response to DNA damage, and high levels are only found in cells expressing mutant forms of p53. After exposure to DENA as a genotoxic compound, high levels of p53 may be induced in cells probably due to stabilization of p53 by phosphorylation by specific kinases such as DNA-activated protein kinase [33]. Moreover, the detection of nontumorigenic wild-type p53 protein (present normally in normal cells) increased the p53 expression levels [34]. Garlic and cinnamon oils succeeded to manage the apoptotic and inflammatory effects of DENA/2-AAF on hepatocytes with downregulation of p53 expression by preventing DNA damage and facilitating DNA repair supporting their competence to control apoptosis in the DENA/2-AAF-mediated HCC model. Therefore, apoptosis favoring therapeutic properties of garlic and cinnamon oils by downregulation of p53 could be one of the important mechanisms of preventing HCC.

Sequence analysis of the three amplicons representing partial sequence of intron 4, exon 5, partial sequence of intron 5, exon 6, exon 7, and partial sequence of intron 7 of rat p53 gene in both control and HCC-induced groups revealed seven genetic mutations in intron 5: five of these were affecting a single base and two were an insertion type. p53 is a highly mutant gene in all cancer types, with a high percent of SNPs are localized in noncoding DNA regions (introns) which involved in transcriptional regulatory mechanisms [35, 36]. Intron sequences play a tremendous role in the regulation of p53 gene expression and DNA-protein interactions, and thus, mutations in those regions may affect these functions by initiating aberrant premessenger RNA splicing, producing mRNA that may be translated into a defective protein [37, 38]. Polymorphisms in the noncoding region of p53 have been suggested to impact on the function or level of expression of p53 and/or increase mutant p53 protein levels as proved by/correlated with IHC analysis of this study. Therefore, the identification p53 gene intron sequence mutations may provide useful tools for the early detection of patients with high risk of cancer development.

## 5. Conclusion

This study substantiates the biochemical, antioxidant, and pathophysiological preventive properties of garlic and cinnamon oils as antihepatocellular carcinoma agents. Taken together, all the results suggest that regular use of these oils may be an attractive and useful therapeutic approach to control proliferation of liver cancer cells; however, further investigations are needed before wide-scale recommendation.

## Figures and Tables

**Table 1:** Gene-specific oligonucleotide primers used in PCR amplification of rat p53 gene exons.

Primer name	Sequence	Gene accession no.	Location	Product size
Exon 5-1F	5-GACCTTTGATTCTTTCTCCTCTCC-3	AH002222.2	1058-1081	265 bp
Exon 5-1R	5-GGGAGACCCTGGACAACCAG-3		1302-1321	
Exon 6-1F	5-GCCTCTGACTTATTCTTGCTC-3	AH002222.2	1323-1343	273 bp
Exon 6-1R	5-CCCAACCTGGCACACAGCTTC-3		1575-1595	
Exon 7-1F	5-CTGTGCCTCCTCTTGTCCCG-3	AH002222.2	1605-1624	188 bp
Exon 7-1R	5-CCACCTTCTTTGTCCTGCCTG-3		1772-1792	

**Table 2:** Body weight and absolute liver weight of rats in different experimental groups (mean ± SE).

Weight (g)	Experimental groups			
	Control	HCC-induced	Garlic oil-HCC	Cinnamon oil-HCC
Body	250.6^a^ ± 10.26	185.2^b^ ± 1.6	234.6^a^ ± 0.75	224.4^a^ ± 5.85
Liver	7.032^b^ ± 0.30	10.18^a^ ± 0.09	7.94^b^ ± 0.34	8.02^b^ ± 0.21

Letters a-b denote significant to nonsignificant differences between the control and other experimental groups. Different letters indicate significant differences between interacting groups at *P* < 0.05.

**Table 3:** Serum biochemical enzyme levels of experimented rats (mean ± SE).

Enzymes	Experimental groups			
	Control	HCC	Garlic oil-HCC	Cinnamon oil-HCC
ALT (IU/l)	66.6^b^ ± 0.40	167^a^ ± 3.39	68^b^ ± 0.71	69.8^b^ ± 0.86
AST (IU/l)	62^b^ ± 1.23	131.6^a^ ± 0.75	61.2^b^ ± 0.56	62.8^b^ ± 1.24
ALP (IU/l)	44.2^c^ ± 1.51	94.8^a^ ± 1.71	47.6^b^ ± 0.51	50.4^b^ ± 0.51
GGT (IU/l)	31.8^b^ ± 1.07	97.4^a^ ± 1.96	33.6^b^ ± 0.81	29.4^b^ ± 0.68

Letters a-c denote significant to nonsignificant differences between the control and other experimental groups. Different letters indicate significant differences between interacting groups at *P* < 0.05.

**Table 4:** GSH, SOD, and MDA in the liver tissue of experimented rat groups (mean ± SE).

Parameter	Experimental groups			
	Control	HCC	Garlic oil-HCC	Cinnamon oil-HCC
GSH (mmol/g tissue)	3.06^a^ ± 0.12	0.54^c^ ± 0.07	1.20^b^ ± 0.114	1.54^b^ ± 0.29
SOD (U/g tissue)	41.2^a^ ± 0.59	4.66^b^ ± 0.31	39.4^a^ ± 0.51	38.8^a^ ± 0.37
MDA (nmol/g tissue)	9.5^c^ ± 0.23	22.04^a^ ± 0.29	10.98^c^ ± 0.66	12.9^b^ ± 0.41

Letters a-c denote significant to nonsignificant differences between the control and other experimental groups. Different letters indicate significant differences between interacting groups at *P* < 0.05.

**Table 5:** Histopathological lesion scoring in the liver in different experimented groups.

Lesion	Experimental groups			
	Control	HCC	Garlic oil-HCC	Cinnamon oil-HCC
Bile duct hyperplasia	0.00^c^ ± 0.00	2.8^a^ ± 0.20	1.2^b^ ± 0.20	1.00^b^ ± 0.00
Degenerative changes	0.00^c^ ± 0.00	3.00^a^ ± 0.00	1.00^b^ ± 0.00	1.2^b^ ± 0.2
Mitotic index	0.00^b^ ± 0.00	4.54^a^ ± 0.28	0.56^b^ ± 0.16	0.58^b^ ± 0.18

Letters a-c denote significant to nonsignificant differences between the control and other experimental groups. Different letters indicate significant differences between interacting groups at *P* < 0.05.

## Data Availability

All data were analyzed and included in the text.
